# Limited Liver or Lung Colorectal Cancer Metastases. Systemic Treatment, Surgery, Ablation or SBRT

**DOI:** 10.3390/jcm10102131

**Published:** 2021-05-14

**Authors:** Meritxell Molla, Julen Fernandez-Plana, Santiago Albiol, Constantino Fondevila, Ivan Vollmer, Carla Cases, Angeles Garcia-Criado, Jaume Capdevila, Carles Conill, Yliam Fundora, Carlos Fernandez-Martos, Estela Pineda

**Affiliations:** 1 Department of Radiation Oncology, Hospital Clinic Barcelona, Barcelona 08036, Spain; MOLLA@clinic.cat (M.M.); CASES@clinic.cat (C.C.); CCONILL@clinic.cat (C.C.); 2 Translational Genomics and Targeted Therapeutics in Solid Tumors, August Pi i Sunyer Biomedical Research Institute (IDIBAPS), Barcelona 08036, Spain; 3 Department of Medical Oncology, Hospital Mutua de Terrassa, Terrassa 08221, Spain; julenfernandez@mutuaterrassa.es; 4 Department of Medical Oncology, Hospital Clinic de Barcelona, Barcelona 08036, Spain; SALBIOL@clinic.cat; 5 Department of General and Digestive Surgery, Hospital Clinic de Barcelona, Barcelona 08036, Spain; CFONDE@clinic.cat (C.F.); FUNDORA@clinic.cat (Y.F.); 6 Department of Radiology, Hospital Clinic Barcelona, Barcelona 08036, Spain; VOLLMER@clinic.cat (I.V.); MAGARCIA@clinic.cat (A.G.-C.); 7 Department of Medical Oncology, Vall d’Hebron University Hospital, Universitat Autònoma de Barcelona, Barcelona 08035, Spain; jacapdevila@vhebron.net; 8 Department of Medical Oncology, Instituto Valenciano de Oncología, Valencia 46009, Spain; carlosfmartos@initiaoncologia.com

**Keywords:** oligometastasis, colorectal cancer, ablation, stereotactic body radiation therapy, liver metastases, lung metastases

## Abstract

The prognosis for oligometastatic colorectal cancer has improved in recent years, mostly because of recent advances in new techniques and approaches to the treatment of oligometastases, including new surgical procedures, better systemic treatments, percutaneous ablation, and stereotactic body radiation therapy (SBRT). There are several factors to consider when deciding on the better approach for each patient: tumor factors (metachronous or synchronous metastases, RAS mutation, BRAF mutation, disease-free interval, size and number of metastases), patient factors (age, frailty, comorbidities, patient preferences), and physicians’ factors (local expertise). These advances have presented major challenges and opportunities for oncologic multidisciplinary teams to treat patients with limited liver and lung metastases from colorectal cancer with a curative intention. In this review, we describe the different treatment options in patients with limited liver and lung metastases from colorectal cancer, and the possible combination of three approaches: systemic treatment, surgery, and local ablative treatments.

We have divided this review in 3 blocks:

1. Percutaneous ablation and stereotactic body radiotherapy of colorectal cancer liver and lung metastases.
2. Systemic treatment of patients with colorectal cancer resectable liver metastases.
3. Systemic treatment of patients with colorectal cancer potentially resectable liver metastases.

## 1. Percutaneous Ablation and Stereotactic Body Radiotherapy of Liver and Lung Metastases from Colorectal Cancer

ESMO guidelines [1] indicate that the selection of the best tools for the treatment of oligometastatic patients should be based on the following:

- The size and localization of the metastases and therefore, access regarding selection of the best treatment method;
- The local control rates achieved (with greater local control for surgery than for the remaining options);
- The invasiveness of the technique;
- The non-tumor-related prognostic considerations and patient-relevant factors as well as patient preferences;
- The local expertise regarding the use of each ablative treatment method;
- Consideration of patient frailty and life expectancy [2].

### 1.1. Percutaneous Ablation

Percutaneous ablation is described as the direct application of energy or chemical therapies to destroy local tumors. The placement of the applicator into the tumor must be carried out under image guidance (CT, MR, US). The procedure can be performed under general anesthesia or conscious sedation.

#### 1.1.1. Types of Percutaneous Ablation

In the last 20 years, diverse technologies have been developed and used for percutaneous tissue image-guided thermal ablation. The three most commonly used types of percutaneous ablation are radiofrequency ablation (RFA), microwave ablation (MWA), and cryotherapy. RFA is a widely extended technique and has been the most evaluated to date [3]. 

RFA is an electric current-based technique that heats the tissue by fractioning electrons at around 400 kHz. The thermal effect is produced by active heating along the device and progressive diffusion of the temperature to the target. An expandable array used together with an electrode of a diameter at least 10 mm larger than the target tumor has been proven to successfully ablate tumors with less than 10% local recurrence [4]. On the other hand, using an array diameter with a margin smaller than 10 mm larger than the target resulted in a poorer 30% local recurrence [5].

MWA is a field-based technology that creates an electromagnetic field around the ablation device in the microwave spectrum ranging from 915 MHz to 2450 MHz, and heats tissues by rotating water molecules, which generates frictional heat. MWA yields a more uniform ablation zone than RFA [6]. 

Cryotherapy kills and damages tumoral cells through a complex combination of diverse mechanisms that occur in the process of tissue freezing and thawing. First, cryotherapy produces protein denaturation and membrane disruption that kills the cells. After that, the production of intra- and extra-cellular ice crystals increases cellular injury and vasoconstriction and occlusion of blood vessels, producing secondary osmotic changes as well as local tissue edema, finally leading to hypoxic tissue injury and coagulative necrosis.

#### 1.1.2. Lung Metastases Ablation

Lung is a frequent location of metastatic disease in around one-third of all cancer patients, and 10–15% of patients with colorectal cancer (CRC) will suffer lung metastatic disease. The majority of lung metastatic patients have multiple lung lesions or metastatic disease present at other, distant organs. Surgical lung metastasectomy has demonstrated good efficacy, leading to a 5-year OS rate in the case of colorectal cancer of 53.5% [7] and 67.8% after R0 resection [8]. However, pulmonary metastasectomy may be contraindicated due to the patient’s condition, including respiratory disabilities, history of previous radiation treatment, or inadequate performance status [9]. Additionally, disease recurrence is expected in more than 50% of patients that undergo colorectal cancer curative resection. 

RFA is the most evaluated technique in the literature. One of the largest studies, of 566 patients presenting 1037 metastases that were treated with RFA, reported survival outcomes comparable to those obtained with surgery [10]. A median OS of 62 months was observed after RFA, and the 5-year OS was 51.5%. Local tumor progression rates per tumor were 5.9%, 8.5%, 10.2%, and 11,0% at 1, 2, 3, and 4 years, respectively. Size of the tumor is described to be a predictive variable of local tumor progression. For that reason, RFA is an option for the treatment of lung metastases smaller than 2 to 3 cm. In the case of oligorecurrent disease, good tolerance of RFA may well offer the possibility of multiple sessions of treatment and delay resumption of systemic treatment.

The study published by Vogl et al. [11], showed better local control in patients with colorectal cancer lung metastases treated with MWA (88.3%) than in patients treated with RFA (69.2%). In the same paper, the authors demonstrated that the local control of tumors located < 5 cm from the hilum was lower than the local control of tumors located > 5 cm from the hilum [11].

The role of cryotherapy is less established. A prospective multicenter trial showed local control of 96.6% and 94.2% at 6 and 12 months, respectively [12]. One-year overall survival rate was 97.5%. Vogl et al. [11] showed no statistical differences between RFA and MWA in terms of time to progression or survival rates. 

In general terms, the largest size of lung metastases > 3 cm is associated with a reduced overall survival [13]. Lack of extrapulmonary metastases and normal carcinoembryonic antigen (CEA) level are independently associated with a favorable prognosis [9]. For the case of non-operable colorectal lung metastases, the published studies suggest an association between RFA and favorable outcome, relatively high survival rates, and good local control.

In patients with lung oligometastasic disease, thermal ablation should be considered if resection is limited due to comorbidity, the extent of the lung parenchyma resection, or other relevant factors (IV, B) [1]. RFA can be used in combination with surgery with the goal of eradicating the visible metastatic sites (II, B) [1].

#### 1.1.3. Liver Metastases Ablation

Surgical resection is nowadays the curative treatment accepted for oligometastatic disease when it is considered technically possible [1]. The evidence for liver ablation is low but it has been reported to have 5-year overall survival rates of up to 50% in selected groups [14]. It requires fewer hospital stays and is associated with less morbidity and mortality than surgery, but its role in the treatment of patients with potentially resectable metastasis is unclear. Several prospective randomized controlled trials are in progress currently to define the efficacy of ablation vs. resection [15,16]. Thermal ablation represents a potentially curative option when surgical treatment is not technically possible because of difficult locations or comorbidities that contraindicate surgery. Ablation also has been applied in unresectable liver disease in conjunction with chemotherapy, improving survival [17]. 

The standard of care for ablation is RFA, but MWA seems to be an alternative. MWA heats up more rapidly and leads to a larger area of necrosis than RFA, but with similar mortality and morbidity [18]. 

In general, lesions suitable for percutaneous ablation are those lesions less than 3 cm in size. For such lesions, the local recurrence rate is around 3% [19,20]. Tumors larger than 3 cm but within 5 cm are associated with impaired overall survival, but there is a consensus that these lesions can effectively be treated by ablation depending on their anatomical position [21]. Thermal ablation is not recommended for liver metastases > 5 cm because the recurrence rate is very high (25–45%). In any case, a safety margin of necrosis greater than 5 or 10 mm is necessary for satisfactory local tumor control. The increase in the safety margins has provided a significantly lower recurrence rate in RFA performed over the last decade compared with earlier outcomes.

There is a general consensus regarding the limit to the number of liver metastases that can be treated. Most centers treat patients with five lesions or fewer, but for some specific cases, up to nine tumors could be considered.

Not all lesions are candidates for treatment with ablation. RFA or MWA are discouraged for lesions closer than 1 cm to the main bile ducts; in these locations, an alternative technique could be irreversible electroporation (IRE). There are some relative contraindications, such as lesions close to the stomach or colon. In these locations, ablation is possible, but requires the help of specialized methods such as hydro-dissection to reduce the associated risk of injury to these structures. An alternative is to perform the ablation laparoscopically. 

The proximity to the vessels is not a contraindication, but it is known that the cooling effect of the flow could reduce the effectiveness of ablation. This cooling effect seems be lower with MWA than RFA.

Only one randomized clinical trial, the CLOCC trial, has demonstrated that aggressive local treatments can prolong PFS and OS in patients with unresectable colorectal liver metastases [22].

### 1.2. Stereotactic Body Radiotherapy (SBRT)

Advances in radiation delivery technology and image guidance have allowed the development of novel radiotherapy techniques including stereotactic body radiotherapy (SBRT). Originally developed by physicians in Sweden as an alternative to surgical resection to treat brain metastases, SBRT is now being used to treat primary and metastatic sites throughout the body, including intrathoracic, liver, adrenal, and bony metastases.

The American Society for Radiation Oncology (ASTRO) defines SBRT as external beam radiotherapy used to deliver a high dose of radiation very precisely to an extracranial target within the body, as a single dose or a small number of fractions [23].

SBRT delivers a high radiation dose to small, well-defined tumor targets while limiting the dose received by surrounding normal tissue. SBRT is a non-invasive treatment and is able to deliver ablative doses to target lesions, leading to increased local lesion control rates with acceptable levels of toxicity.

#### 1.2.1. SBRT Technique

SBRT is a noninvasive local therapy. Several different mechanisms are now available such as linear accelerator, helical tomotherapy, or specifically dedicated stereotactic machines, all of which effectively deliver highly focused radiation.

For lesions that exhibit intrafraction motion due to normal respiration, e.g., lung and liver lesions, motion management techniques are necessary. These techniques include respiratory correlated 4-dimensional CT, breath-hold CT using active breathing control with breath hold deep inspiration, and free breathing CT using abdominal compression.

**Figure 1:**
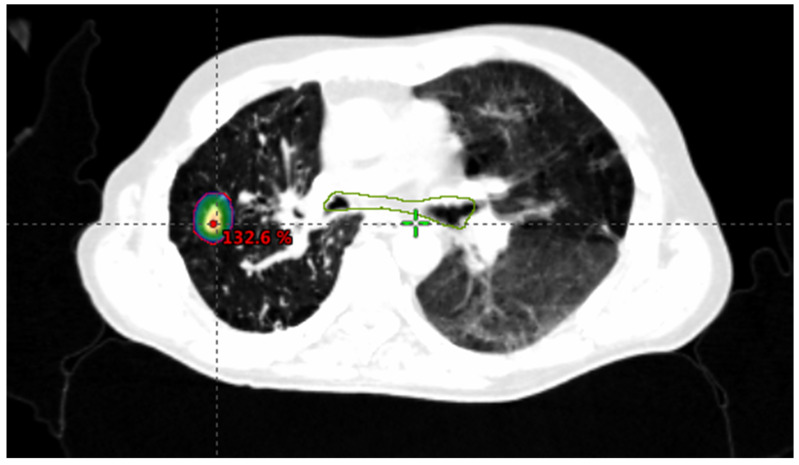
Lung metastases from CRC. Planning treatment: Dose: 60 Gy in 3 fractions. DBE: 150 Gy.

**Figure 2:**
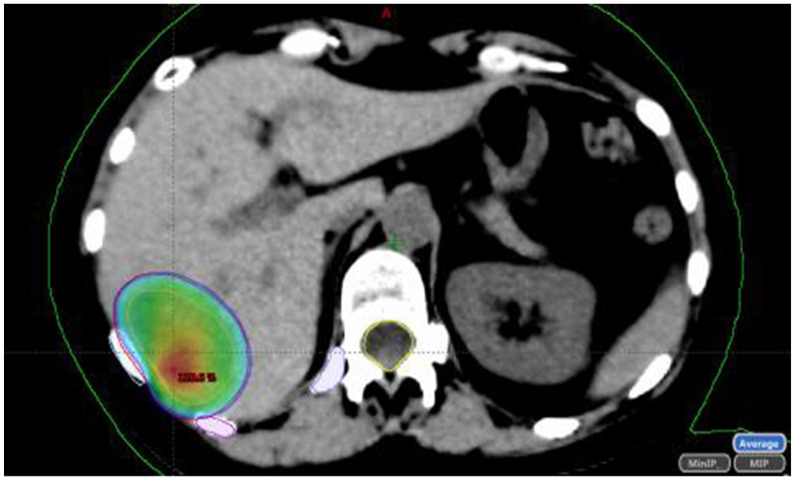
Liver metastases from CRC. Planning treatment. Dose: 55 Gy in 5 fractions. DBE: 116 Gy.

First, axial CT images are obtained along the region of interest. For all the treated lesions, the gross tumor volume is defined as the visible tumor on the CT scan with the help of MRI and/or PET available images. A margin of 2–5 mm will be added depending on the site of disease and immobilization device to define the planning target volume. A 5 mm margin may be used for lung (Figure 1) and liver tumors (Figure 2).

Treatment setups using a margin will assure a reproducible positioning and will be verified by the image-guidance protocol. Image guidance for treatment verification and delivery is mandatory for all fractions with on-line corrections prior to each fraction. For liver metastases, fiducial placement, generally via ultrasound, is used in some centers to improve treatment verification.

#### 1.2.2. SBRT Dose

SBRT doses were prescribed and delivered depending on the treated metastatic site. For some of the metastatic sites (liver, lung, and lymph node), the prescribed number of fractions was chosen according to the volume of the metastasis and its proximity to dose-constraining normal tissues. The normal tissue dose constraints were based on international guidance publications [24,25]. Dose prescriptions were based on experiences gained in primary non-small cell lung cancer for the case of pulmonary metastases, and on maximally tolerant doses for the organs at risk in the case of liver irradiation. In the cases where dose constraints were not achievable, the target dose was reduced accordingly.

Treatment was delivered following an alternate day scheme. There are numerous publications that report toxicity after SBRT [23,26]. 

Various dose-fraction models have been proposed to predict the effectiveness of radiotherapy doses and fractionation regimens. Varying dose-fractionated schedules have been utilized in the stereotactic treatment of oligometastatic disease. The optimal regime has yet to be defined. However, ablative radiotherapy doses require a higher biologically effective dose (BED) than do most conventionally fractionated regimens. BED is a measure of the effectiveness of different dose fraction regimens, to allow comparison of different doses or doses per fraction. Regimens resulting in a BED larger than 100 Gy equivalent dose in 2 Gy fractions (EQD2) would be deemed ablative.

Typical dose schedules range from 3–8 fractions delivering 45–60 Gy for lung and liver metastases.

#### 1.2.3. Criteria for Determining SBRT Suitability

Patients suitable for radical treatment of oligometastases are described at the beginning of this chapter.

To be considered suitable for SBRT, lesions must remain at or below 5 cm in size. Parameters for the measurement of lesions will be in accordance with standard RECIST v1.1 procedures. In a large patient cohort from a multi-institutional database and focusing on colorectal cancer (CRC) with lung and liver metastases, radiation dose, tumor size, and pre-SBRT chemotherapy could be identified as predicted factors for local control after SBRT [27].

In liver metastases, compared with radiofrequency (RFA), SBRT has the advantage of accessing metastases that are more challenging to reach with RFA (e.g., periampullary, perihilar, or subcapsular locations). Additionally, SBRT appears better suited in treating tumors adjacent to vasculature and in large tumors (especially > 4 cm) [28]. In a phase II trial of SBRT [29], the authors analyzed 42 patients that presented liver metastases from colorectal cancer not amenable to surgery or RF. The main reasons to avoid surgery were, age and/or co-morbidities (19 patients) and recurrence after previous surgery (17 patients), and the main reason to avoid RF were lesions larger than 3 cm (20 patients) and lesions close to vascular, biliary, or gastrointestinal structures (18 patients). With a median follow-up of 24 months (range 4–47), local control was 91% with median overall survival of 29.2 ± 3.7 months. No patients experienced radiation- induced liver disease or toxicity grade ≥ 3. SBRT appears to be a feasible alternative for the treatment of colorectal liver metastasis in patients unsuitable for surgery or RF.

#### 1.2.4. SBRT Outcomes

A review of published data on SBRT in oligometastases [30] showed that the most reported metastases were liver and lung metastases. There is wide heterogeneity in reported populations, which consist basically of patients in retrospective and prospective cohort studies. Most of these studies report that 72–92% local control can be achieved for lung metastases [31] and of 60–90% for liver metastases at 2 years [32]. A recent meta-analysis [33] including 943 patients with ≤5 sites of extracranial disease, and SBRT administrated in ≤8 fractions with ≥5 Gy per fraction, reported that colorectal cancer was the second most common primary site (16.6%). Rates of acute and late grade 3–5 toxic effects were less than 10%, the estimated rate of 1-year local recurrence was 94.7%, and the estimated 1-year overall survival was 85.4%. The authors suggested that SBRT is well tolerated and of clinical benefit.

We have recent data available from a multicenter randomized phase II trial to assess the impact of a comprehensive oligometastatic SBRT treatment on overall survival, oncologic outcomes, toxicity, and quality of life in patients with up to five metastatic cancer lesions and a controlled primary tumor, compared to standard of care treatment alone [34]. From February 2012 to August 2016, 99 patients were enrolled. Eighteen (18.2%) patients had colorectal primary cancer. A significant statistical prolongation of progression-free survival from 6 months to 12 months was seen in the SBRT arm. An improvement of overall survival from 28 month to 41 months was also seen. 

In selected colorectal patients, SBRT is increasingly adopted to treat limited liver or lung metastases. In a systematic review, Petrelli et al. [35] analyzed a total of 656 patients with CRC liver oligometastases of about 3 cm in size, generally pre-treated with systemic therapies. SBRT resulted in long-term local control and progression-free survival at 2 years (59% and 56%, respectively).

Data from two retrospective SBRT databases of 500 liver and lung metastatic lesions from colorectal cancer with a reported median follow-up of 12.1 months showed that local relapses were recorded in 31.3% and 10% of liver metastases and lung metastases, respectively. A survival benefit was observed in the cases where local control of individual metastases had been achieved [27].

A multidisciplinary team should work together to identify patients who will benefit from local therapies. SBRT is a non-invasive technique with low and limited toxicity and encourages local control of disease and good survival rates. SBRT is an effective treatment option for patients with oligometastatic colorectal cancer disease.

Percutaneous ablation and stereotactic body radiotherapy of liver and lung metastases from colorectal cancer.

Recommendations:

- Evaluate these patients with a multidisciplinary team of experts in colorectal cancer.
- Local ablative treatments or SBRT must be considered to treat limited lung and liver CRC M1 smaller than or equal to 5 cm.
- RFA is preferred in CRC M1 smaller than 3 cm. MWA could be considered in CRC M1 ≤ 5 cm.
- There is no maximum limit on the number of lesions to treat with ablative treatments or SBRT, but the consensus from most studies recommends a maximum of 5 lesions.
- Consider SBRT to treat lung and liver M1 close to vascular, biliary, or gastrointestinal structures;, laparoscopic ablative treatments also could be considered.
- Take into account clinical and molecular tumor prognostic factors, patient preferences, patient comorbidities, and center experience.

## 2. Treatment of Patients with Resectable Liver Colorectal Metastases (Liver M1: Number <4 and Size <5 cm)

The good prognosis subgroup is defined by the presence of less than four liver metastases, all smaller than 5 cm. In this context, the role of adjuvant chemotherapy after liver surgery has been studied in a few randomized studies. All of these trials were hampered by the limited numbers of patients recruited, differences in the definition of the population (number of liver nodules, resectability criteria), the chosen primary endpoint (DFS, PFS or OS), and the heterogeneity in stratification criteria. Langer et al. [36] compared metastasectomy alone vs. surgery followed by systemic 5-FU/LV treatment in 107 colorectal cancer (CRC) patients who underwent resection of hepatic metastases. Although DFS and OS were better in the chemotherapy arm (4-year DFS 45% vs. 35%, and 4-year OS 57% vs. 47%), the trial was prematurely closed because of slow accrual and failure to reach statistical significance. In a multicenter trial [37], 173 CRC patients were randomized after liver resection of metastases to receive adjuvant systemic chemotherapy with 5-FU/LV or to a control arm without adjuvant therapy. In this study, also closed prematurely because of slow accrual, an improvement was observed in DFS for patients treated with adjuvant 5-FU/LV compared to the control arm (24.4 months vs. 17.6 months, *P =* 0.028) but without significant differences in OS (5-year OS: 51.1% vs. 41.1%). A pooled analysis of these clinical trials showed a minimal statistical significance in favor of the 5-FU/LV-based adjuvant chemotherapy regimen (PFS: 27.9 vs. 18.8 months, HR = 1.32; 95% CI, 1.00–1.76; *P* = 0.058; OS 62.2 vs. 47.3 months, HR = 1.32; 95% CI, 0.95–1.82; *P* = 0.095), and adjuvant 5FU/LV chemotherapy was associated with both PFS and OS in multivariable analysis [38]. In another phase III trial [39], 180 CRC patients were randomized after liver resection to receive adjuvant UFT/LV or surgery alone. The 3-year DFS was 38.6% in the chemotherapy group and 32.3% in the surgery group (*P =* 0.003), but no significant difference in OS was observed (3-year OS 82.8% vs. 81.6%, *P =* 0.41). Recently, a Japanese group presented results of the JCOG0603 at ASCO 2020, in which patients with resected liver metastases were randomized to observation or adjuvant chemotherapy with FOLFOX6m × 12 cycles, in a phase III trial. This trial demonstrated a benefit of adjuvant FOLFOX6m for disease-free survival (DFS 1.7 years vs. 4.3 years, HR 0.67 (0.5–0.92)) but no benefit for overall survival (5-year OS 83% vs. 71%, HR 1.25 (0.78–2)) in this subgroup of patients [40]. Adjuvant treatment with irinotecan in patients with CRC after liver surgery was not shown to be superior to 5FU/LV in a phase III study [41].

Kemeny et al. [42,43] randomized 156 patients who underwent resection of hepatic metastases from CRC to systemic chemotherapy alone (5-FU with or without LV) or to HAI-FUDR plus systemic chemotherapy. After a median follow-up of 10 years, PFS was significantly better in the combined-therapy group than in the monotherapy group (31.3 vs. 17.2 months, *P* = 0.02). Hepatic arterial infusion (HAI) with or without chemotherapy significantly increased DFS compared to chemotherapy alone in two of three randomized studies [44,45]. However, no statistically significant benefit was observed for OS (68.4 vs. 58.8 months, *P =* 0.10). 

The role of perioperative chemotherapy with FOLFOX4 was evaluated in the EORTC intergroup trial 40,983 (EPOC). This study clearly demonstrated that perioperative FOLFOX4 therapy (6 cycles before surgery and 6 cycles after) significantly improved PFS compared to surgery alone in CRC patients with resected liver metastases [46]. Median PFS was better in the FOLFOX arm, at 18.7 months compared to 11.7 months in the control arm, with a 3-year PFS of 35.4 versus 28.1% (HR of 0.79; 95% CI 0.62–1.02). Despite demonstrating no benefit in OS with long term follow-up [47], this strategy is widely used in daily practice because it maintains the benefit in PFS and helps avoid unnecessary resection in patients with early progressive disease (8–10%), and for these reasons may be considered the standard of care. 

A combination of chemotherapy with biological targeted agents improves the clinical outcomes of metastatic CRC, whereas there is no evidence supporting their use after resection of metastases even in the adjuvant setting. The new EPOC study randomized 272 wildtype *KRAS* patients with resectable CRC liver metastases to chemotherapy with cetuximab vs. chemotherapy alone. PFS was significantly shorter in the cetuximab group than in the chemotherapy alone group ( 14.1 vs. 20.5 months, HR 1.48; *P* = 0.03) [48,49]. The HEPATICA study randomized 79 patients with resectable CRC liver metastases to CAPOX with or without bevacizumab and no differences were found in PFS or OS [50] ( Table 1).

Treatment of patients with resectable liver colorectal metastases. Recommendation:

- A perioperative schedule with FOLFOX is recommended in this population of patients.
- Combinations of FOLFOX with bevacizumab or anti-EGFR cannot be recommended based on RCTs.
- Stratification criteria in prospective RCTs should consider the number of liver nodules (1 vs. 2–4) and the DFI (disease free interval) <12 vs. >12 months.
- In elderly and frail patients, consider SBRT or local ablative treatments alone or in combination with surgery. Take into account center experience and patient preferences.
- Evaluate all of these patients with a multidisciplinary team of experts in colorectal cancer.

## 3. Treatment of Potentially Resectable Liver Metastases from Colorectal Cancer (Liver M1: Number >4 or Size >5 cm)

The efficacy of conversion chemotherapy has increased in recent years. Conversion chemotherapy represents an opportunity for downsizing tumor disease, and thus, convert patients with initially unresectable disease to resectable disease [51]. Although survival following conversion chemotherapy and surgery is lower than survival achieved by patients who are candidates for primary liver resection, it is still clearly better than not having performed liver surgery at all. 

The current description of resectability includes the potential for complete resection with tumor free margins (R0 resection, 1 mm minimum margin) with viable vascular inflow, outflow, and biliary drainage and a functioning liver remnant volume of 25–30% [52].

Around 20–30% of newly diagnosed mCRC patients have synchronous liver metastasis and there is no standard of care for treating it. Although synchronous liver metastasis from CRC is traditionally treated with a two-stage resection, simultaneous resection of liver metastases and primary tumor is possible in selected cases. Moreover, resection of liver metastasis first (reverse treatment) is probably the best option when liver disease is predominant and primary tumor is pauci-symptomatic [53]. 

Portal vein embolization, two-staged hepatectomies, and hepatectomies with radiofrequency ablation (RFA) are strategies that can help to achieve an R0 resection.

Conversion chemotherapy results in a greater resectability rate, higher probability to perform surgery in patients with initially unresectable tumors, and a higher survival rate. 

Some of the trials evaluating the role of conversion treatment strategy had different patient selection and resection criteria. Moreover, only a few of these trials were randomized controlled trials, which made it difficult to select the best conversion therapy option. Different schedules and biological agents have been evaluated in this field.

A cetuximab-containing schedule was first evaluated in this setting in the CELIM trial. In this trial, patients with ≥5 liver metastases and/or technically unresectable CRC liver metastases were treated with FOLFOX/cetuximab or FOLFIRI/cetuximab and, after systemic treatment, were evaluated for resectability. A total of 111 patients were randomized to FOLFIRI or FOLFOX, both with cetuximab. A 70% response rate was achieved in patients with KRAS exon 2 wild-type disease, which led to a promising 33% R0 resection rate. The ITT (intention to treat) population reached a 35-month median OS, and patients with R0 resection achieved a 53-month median OS. According to the retrospective review, rates of resectability significantly increased from 32% to 60% after cetuximab plus chemotherapy. However, these valuable results, were not compared with a non-cetuximab controlled arm and hence, no clear conclusions can be drawn regarding the benefit of adding cetuximab based on the CELIM trial [54,55]. Likewise, the POCHER trial assessed chemotherapy plus cetuximab in this setting. This was a non-randomized single-arm trial where 43 patients with unresectable metastases were treated with Chrono-IFLO plus cetuximab. Cetuximab plus this chemotherapeutic scheme (Chrono-IFLO) achieved a 60% R0 resection rate. ORR was 79% and median OS, 37 months with a 68% 2-year OS (80% 2-year OS in resected patients) [56].

Ye et al. conducted a Chinese phase II prospective randomized controlled trial assessing the role of adding cetuximab to a conversion treatment schedule [57]. In this trial, 138 patients with KRAS exon-2 wild-type liver limited disease were included. Patients received a cetuximab-containing chemotherapy (mFOLFOX6/FOLFIRI) or chemotherapy alone. The cetuximab-containing chemotherapy arm was associated with an increase in R0 resection rate, moving from 7% in the control arm up to 26% in the cetuximab arm. Furthermore, 29% of patients in the cetuximab-containing arm became eligible for resection compared to 13% in the control arm. As expected, patients in either treatment arm undergoing surgery achieved a longer median survival time than those who did not undergo surgery.. 

Bevacizumab also has been assessed to improve conversion treatment schedules. In a randomized phase II study, the addition of bevacizumab to HAI plus chemotherapy in CRC patients after liver surgery showed no improvement in PFS or OS, with an increase in biliary toxicity [58]; therefore, this combination is not recommended. Gruenberger et al. conducted one of the first trials assessing a bevacizumab-containing chemotherapy as a conversion treatment [59]. They conducted a non-randomized single-center phase II trial. Fifty-six patients at high risk of early recurrence were treated with CAPOX + bevacizumab. ORR was achieved in 73% (41 patients). Similarly, the BOXER trial recruited 46 patients with poor-risk CRC liver metastases not selected for upfront resection [60]. In this multicenter, single arm, phase II trial, patients were treated with CAPOX plus bevacizumab and achieved a 78% ORR. In terms of resectability, patients with nonsynchronous, initially unresectable disease were resected in 40% of cases. Moreover, synchronous resectable CLM underwent liver resection in 67% of the cases. In like manner, the OLIVIA trial, a randomized controlled European phase II trial, assessed a bevacizumab-containing chemotherapy for unresectable CRC liver metastases. Patients were randomized to receive bevacizumab plus either FOLFOXIRI or mFOLFOX6. Resection rates were 61% or 49%, respectively. R0 resection rates were 49% and 23%, respectively. Overall tumor response rates were 81% with bevacizumab-FOLFOXIRI and 62% in the bevacizumab-mFOLFOX6 arm. Although FOLFOXIRI-bevacizumab resulted in a highly active schedule, the intensification was largely due to the addition of a third chemotherapy agent. As bevacizumab was included in both arms, its role in this setting still remains unclear [61]. 

In all of these trials, the intensification schedule/arm achieved high ORR, which led to high R0 resection rates for these surgically challenging, potentially resectable patients. Sidedness has been described as a prognostic and predictive marker for mCRC [62], with better survival results for left-sided tumors than for right-sided tumors. According to two meta analyses [63,64], the predictive effect of tumor side when antiangiogenic and anti-EGFR were compared in terms of ORR, led to the conclusion that anti-EGFR combination treatment not only achieves higher ORR in left-sided tumors, but also in right-sided ones. If the goal is ORR and resectability, then for RAS wild-type patients, an anti-EGFR-combination schedule seems to work better than antiangiogenic combinations, regardless of sidedness. The deepness of the response has also been reported to be higher for anti-EGFR combinations when compared to antiangiogenic combinations [65,66]. However, a FOLFIRINOX plus bevacizumab and a cetuximab-doublet-containing schedule have not been compared (Table 1). 

Treatment of potentially resectable liver metastases from colorectal cancer. Recommendations:

- A highly active regimen in terms of ORR and tumor shrinkage is recommended for patients with potentially resectable CRC liver metastases.
- For RAS wild-type patients, an anti-EGFR combination schedule is recommended regardless of sidedness, if conversion is the goal.
- For RAS mutant patients, a bevacizumab combination-schedule is recommended regardless of sidedness, if conversion is the goal. For fit patients, FOLFIRINOX-bevacizumab is the preferred schedule.
- In elderly and frail patients consider SBRT or local ablative treatments alone or in combination with surgery if good response is achieved with systemic treatment. Consider patient preferences and center experience.

## 4. Conclusions

**Figure 3:**
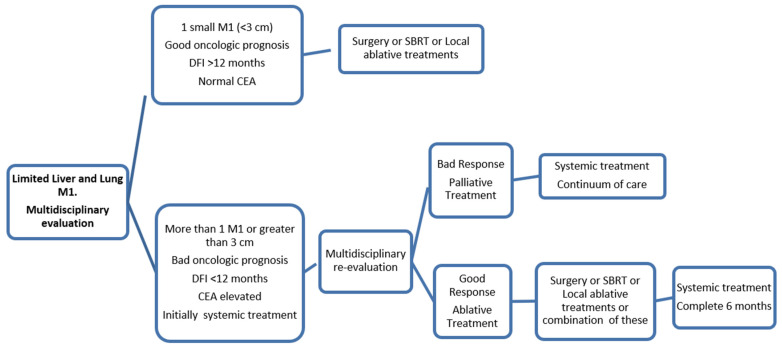
Treatment algorithm in limited liver and lung CRC metastases.

Patients with liver and lung limited metastases from colorectal cancer can be treated with a curative intention, and this context has become more frequent in recent years. New advances such as SBRT or local ablative therapies have become important factors for better palliation, to prolong survival and sometimes to cure patients in this context. This population of colorectal patients must always be evaluated by a colorectal cancer multidisciplinary board of experts in the field, including radiotherapeutic oncologists and interventional radiologists from tertiary hospitals. However, few randomized trials have evaluated the efficacy of these local approaches, and most of the data are retrospective. Probably within the next few years, we will have more prospective data to support these techniques with more robustness. It is crucial to evaluate multiple factors when choosing the best treatment for each patient: tumor prognostic factors (disease-free interval, CEA level, RAS or BRAF mutations, number and size of metastases), patient factors (frailty and comorbidities), anatomic characteristics of M1 (size and location), center experience, and patient preferences (Figure 3). As we described in this review, the role of chemotherapy (alone or in combination with targeted therapy) is well-established in resectable or potentially resectable liver metastases. However, the role of chemotherapy in the setting of resected CRC lung metastases is not well-defined. Given the lack of robust data to support the use of postoperative chemotherapy for resected CRC lung metastases, that role should be evaluated in the context of a randomized controlled trial. 

## Figures and Tables

**Table 1:** ESMO-Magnitude of Clinical Benefit Scale (ESMO-MCBS) of studies with liver limited colorectal cancer patients.

Author	N	Treatments	HR PFS/OS. <0.8	Adequate Control Arm	Any Change in Primary End-Point or Sample Size	Achieved Pre-Specified Objective	Quality of Clinical Design	ESMO. MCBS/PFS	ESMO. MCBS/OS	ESMO/MCBS 1.1
**Randomized clinical trials in CRC with liver limited M1 (<4 M1)**										
Portier, JCO 2006	173	S vs. S plus 5FU/LV	0.66/0.73/0.9	1	0	0	1 of 3	B	A	A
Mitry, JCO 2008	278	S vs. S plus 5FU/LV	1.32/1.32/1	1	1	NA	NA	B	A	A
Hasewaga, Plos One 2016	180	S vs. S plus UFT/LV	0.56/0.8/0,7	1	1	0	2 of 3	A	C	A
Nordlinger, Lancet Oncol 2013	364	S vs. S plus FOLFOX	0.79/0.88/0.89	1	1	0	2 of 3	B	B	B
Ychou, Ann Oncol 2009	321	S plus FU/LV vs. S plus FOLFIRI	0.89/1.09/0.81	0	0	0	0 of 3	C	C	C
Bridgewater JA. Lancet Oncol 2020	257	S plus FOLFOX vs. S plus FOLFOX/CET	1.17/1.45/0.8	1	0	0	1 of 3	C	C	C
Snoeren N. Neoplasia 2017	79	S plus CAPOX vs. S plus CAPOX plus BEV	NA	0	0	0	0 of 3	C	C	C
Kanemitsu. ASCO 2020	300	S vs. S plus FOLFOX	0.67/1.25/0.53	1	1	1	3 of 3	B	C	B
**Randomized Clinical trials in CRC with liver limited M1 (≥4 M1)**										
Ye LC, JCO, 2013	138	CHT plus S vs. CHT/CET plus S	0.6/0.54/1.11	1	NA	1	1 of 3	A	A	A
Gruenberger, Ann Oncol 2015	80	FOLFOX/BVZ plus S vs. FOLFOXIRI/BVZ plus S	0.43/0.35/1.22	0	1	NA	1 of 3	A	NA	A
**Adding RF or SBRT to chemo**										
Ruers T. JNCI 2017	119	FOLFOX vs. FOLFOX plus RF ± S	0.57/0.58/0.98	1	0	1	2 of 3	3	3	3
Palma, Lancet 2019	99	CHT vs. CHT plus SBRT	0.47/0.57/0.82	1	1	1	2 of 3	3	4	4
